# Lactic acid bacteria strains selected from fermented total mixed rations improve ensiling and *in vitro* rumen fermentation characteristics of corn stover silage

**DOI:** 10.5713/ab.21.0461

**Published:** 2022-01-05

**Authors:** Kailang Huang, Hongwei Chen, Yalu Liu, Qihua Hong, Bin Yang, Jiakun Wang

**Affiliations:** 1 Institute of Dairy Science, College of Animal Sciences, Zhejiang University, Hangzhou, 310058, China; 2 The Experimental Teaching Center, College of Animal Sciences, Zhejiang University, Hangzhou, 310058, China

**Keywords:** Corn Stover Silage, Ensiling Characteristics, *In vitro* Rumen Fermentation, *Lactobacillus*, Metataxonomic Analysis

## Abstract

**Objective:**

This study identified the major lactic acid bacteria (LAB) strains from different fermented total mixed rations (FTMRs) via metataxonomic analysis and evaluated the ability of their standard strain as ensiling inoculants for corn stover silage.

**Methods:**

The bacterial composition of eight FTMRs were analyzed by 16S rDNA sequencing. Corn stover was ensiled without LAB inoculation (control) or with 1×10^6^ cfu/g LAB standard strain (*Lactobacillus vaginalis*, *Lactobacillus reuteri*, *Lactobacillus helveticus*, or *Lactobacillus paralimentarius*) selected from the FTMRs or 10 g/t commercial silage inoculant (CSI) around 25°C for 56 days. For each inoculation, a portion of the silage was sampled to analyze ensiling characteristics at time intervals of 0, 1, 3, 7, 14, 28, and 56 days, gas production (GP), microbial crude protein and volatile fatty acids as the measurements of rumen fermentation characteristics were evaluated *in vitro* with the silages of 56 days after 72 h incubation.

**Results:**

*Lactobacillus* covered >85% relative abundance of all FTMRs, in which *L. pontis*, *L. vaginalis*, *L. reuteri*, *L. helveticus*, and *L. paralimentarius* showed >4% in specific FTMRs. CSI, *L. helveticus*, and *L. paralimentarius* accelerated the decline of silage pH. Silage inoculated with *L. paralimentarius* and CSI produced more lactic acid the early 14 days. Silage inoculated with *L. paralimentarius* produced less acetic acid and butyric acid. For the *in vitro* rumen fermentation, silage inoculated with CSI produced more potential GP, isobutyric acid, and isovaleric acid; silage inoculated with *L. helveticus* produced more potential GP and isovaleric acid, silage inoculated with *L. paralimentarius* or *L. reuteri* produced more potential GP only.

**Conclusion:**

The standard strain *L. paralimentarius* (DSM 13238) is a promising ensiling inoculant for corn stover silage. The findings provide clues on strategies to select LAB to improve the quality of silage.

## INTRODUCTION

Corn stover is one of the most abundant seasonal straw resources in the world. It is currently widely used as ruminant feed in animal husbandry. However, epiphytic undesirable microorganisms make corn stover difficult to store, and increase the risk of undesirable microbial metabolites in milk.

Ensiling is a technology commonly applied on farms to preserve animal feed for off- season use and improve feed palatability. During the anaerobic ensiling process, epiphytic lactic acid bacteria (LAB) transforms water-soluble carbohydrates (WSC) of feed into lactic acid, thereby reducing the pH of feed to around 4.0, which prevents the growth of some undesirable microorganisms, such as yeasts, molds, and other aerobic bacteria [1], and the lactic acid enhances feed palatability [2]. Benefit from the faster of pH reduction, the smaller storage failure, exogenous LABs are recommended as additives to accelerate the fermentation of WSC of corn stover into lactic acid. However, compatibility between the corn stover and the inoculated LABs influence ensiling characteristics [3], To overcome the compatibility, the mixture of homofermentative, or homofermentative and heterofermentative LABs are commonly used. In fact, comparing to legumes, the epiphytic microorganism community and the higher WSC concentration, let the grasses be easier to preserve and more stable [4,5]. Undoubtedly, the unspecific mixture LAB inoculation increases the cost in farm.

Lactic acid bacteria are the preferred probiotics, researchers and companies from so many countries are committed to building the bank for LAB strains, some effective LAB strains have been selected from forage crops [6], grasses [7,8], and nature-fermentative silages [9,10]. However, fermented total mixed ration (FTMR), a fermented feed produced via the anaerobic fermentation of total mixed ration, which is widely used to improve the quality and extend the storage time of the feed due to its high aerobic stability has been really ignored. In addition, although the screened LAB strains have been published, they are still private and cannot be applied in farm before commercialization [10,11].

We hypothesized that the terminal dominant bacterial species in FTMRs, especially the roughage including some grasses might improve the quality of corn stover, and their standard strain could be used as a candidate additive for corn stover ensiling. Therefore, the major LAB strains in eight FTMRs were identified using metataxonomic analysis and their potential as ensiling inoculants for corn stover were evaluated by the characteristics of ensiling and *in vitro* rumen fermentation. The selected standard strains in our study can be directly recommended to farms because of the availability of the standard strains.

## MATERIALS AND METHODS

All procedures were approved by the Animal Care and Use Committee of Zhejiang University (Hangzhou, China) and were in accordance with the university’s guidelines for animal research.

### Identification and selection of LAB from FTMRs

Eight FTMRs, one from TMF Center of Sanwa Industries, Japan (Jap1), mainly including brewers grains (beer, wet), timothy, cynodon dactylon, and oat grass; one from Yukijiroshi TMR Center, Japan (Jap2), mainly including soybean curd residue, brewers grains, sugar beet residue, used tea leaves, and beet meal; and six from Nbdg YOYOU Co., Ltd., Ningbo, China (Chi1 to Chi6), including soybean, corn, corn husk, wheat bran, brewer grains, dry peanut straw, and fresh rice straw with different number of wrapping layers. Two kg fresh weight each, were collected in vacuum plastic polyethylene bags, transported on ice, and stored at −20°C in a freezer.

For the molecular analysis of the microbial communities of eight FTMRs, genomic DNA was extracted from the FTMR samples using the cetyltrimethylammonium bromide method described by Gagen et al [12] with a bead-beater (Biospec Products, Bartlesville, OK, USA) after thorough grinding into a powder with liquid nitrogen. Each FTMR sample was divided into four replicates to extract DNA. The DNA was purified using a DNA purification collection tube (B615005; Sangon Biotech Co., Ltd., Shanghai, China). The amplicon library of the V3–V4 hypervariable region of the 16S rRNA gene was prepared from each of the DNA samples using the 341F/806R primer set and sequenced using a 2×250 bp paired-end protocol on an Illumina MiSeq platform (Shanghai Hanyu Bio-Tech Co., Ltd., Shanghai, China).

The raw sequences were demultiplexed, quality-filtered, and analyzed using Mothur v.1.32.1 [13]. Briefly, the paired reads were merged to form single sequences using Mothur [14], and sequences having a length less than 200 bp, continuously repeated bases greater than 8, or ambiguous bases were filtered out. The remaining sequences were dereplicated and aligned with the Greengene database [15], and then possible chimeras were identified and removed using UCHIME [14]. The quality-checked sequences were classified using the Greengene database [15] and clustered into operational taxonomic units at an identity threshold of 97%. The sequenced data reported in the current study have been deposited in the National Center for Biotechnology Information (NCBI) database (Accession No. PRJNA715708).

### Preparation of laboratory silage

Corn was grown in Shandong Province, China. The stover was collected after the harvest of cobs, air-dried on the ground, and kneaded to 50 mm in length with a straw rubber (9RS-1; Xintiandi Prataculture Co. Ltd., Xian, China), and the dry matter (DM) content, crude protein (CP) was determined by the method 924.05, 988.05 of the Association of Official Analytical Chemists, respectively. The water soluble carbohydrate (WSC) was determined by colorimetric method [16]. Approximately 500 g of chopped stover (approximately 92% DM, 9.7% WSC of DM, 3.3% CP of DM) in each of the four replicates was sprayed and mixed with different types of LAB inoculants: i) *L. vaginalis* (DSM 5837), ii) *L. reuteri* (DSM 20016), iii) *L. helveticus* (CGMCC 1.1877), iv) *L. paralimentarius* (DSM 13238), v) no inoculant (control), or vi) commercial silage inoculant (CSI; Baolai-leelai Bio-tech Company, China). The four *Lactobacillus* strains were acquired from the Key Laboratory of Dairy Biotechnology and Engineering, Education Ministry of China, Inner Mongolia Agricultural University, Hohhot, Inner Mongolia, China [17]. The CSI was composed of *Pediococcus acidilactici* (≥1.0×10^9^ CFU/g) and *L. plantarum* (≥3.0×10^6^ CFU/g) and their metabolites. Inoculants were dissolved in distilled water and sprayed into the corn stover to let the initial silages have a moisture content of 65%, and inoculated *Lactobacillus* sp. and CSI calculated at 1×10^6^ CFU/g and 10 g/t wet silage, respectively. The control treatment was sprayed with an equal amount of distilled water. After the corn stover was thoroughly mixed with the inoculant, about 700 g of wet corn stover in each of four replicates was vacuum packed into polyethylene silo bags (30 cm length and 25 cm width) and stored at room temperature (approximately 25°C).

### Analysis of the ensiling characteristics

All of the silages were sampled at day 0, 1, 3, 7, 14, 28, and 56 during ensiling. The bag was opened using a scissor, twenty gram of wet silage per bag were collected immediately, then the bag was re-vacuumed by a vacuum sealer to make sure the compaction and anaerobic fermentation of remaining silage. Each sample was homogenized with 180 mL of sterile distilled water using a grinder (BL25b12; Midea Group, Foshan, China) for 2 min and then filtered through four layers of gauze to obtain the filtrate for pH and chemical analysis. The pH was immediately measured using a glass electrode pH meter (Starter 300; Ohaus Instruments Co. Ltd., Shanghai, China). Volatile fatty acids (VFAs), including acetic acid, propionic acid, butyric acid, isobutyric acid, valeric acid, and isovaleric acid, were measured using gas chromatography (GC-2010; Shimadzu Corp., Kyoto, Japan). Lactic acid and NH_3_-N were detected using a lactic acid assay kit (A019-2-1; Nanjing Jiancheng Bioengineering Institute, Nanjing, China) and the colorimetric method of Chaney and Marbach [18], respectively.

### *In vitro* rumen fermentation

Ten grams of silage from each bag was sampled after 56 days of ensiling, oven-dried, and ground to pass through a 0.425 mm sieve. Four samples from the same treatment group were combined in equal parts for *in vitro* ruminal fermentation following the procedure described by Menke et al [19]. The DM content of the dried silage mixture was determined by the AOAC method 924.05.

*In vitro* fermentation studies were conducted in triplicate, and three blanks were included simultaneously. Ruminal fluid was collected from three adult Hu sheep fed a total mixed ration (roughage/concentrate = 70:30, the roughage, including equal amounts of Chinese wild rye and alfalfa), 1 h before the morning feeding. Ruminal fluid was mixed with prewarmed buffered medium at a 1:9 ratio at 39°C. A total of 50 mL of incubation medium was transferred into a 120 mL serum bottle that was preloaded with the dried silage mixture (0.5 g). The bottles were sealed with butyl rubber stoppers, secured with an aluminum crimp, and incubated at 39°C. All of the above processes were performed under anaerobic conditions maintained by continuous infusion of carbon dioxide (CO_2_) gas. The gas pressure in each bottle was recorded at 3, 6, 9, 12, 24, 36, and 72 h using a pressure sensor (Ruyi, Shanghai, China). After 72 h of incubation, the bottles were placed on ice to stop the fermentation, and the fluid was sampled and preserved at −20°C for the analysis of VFA, NH_3_-N, and microbial crude protein (MCP). The VFA and NH_3_-N were determined by the above methods, and MCP was determined using the method described by Mi et al [20].

### Statistical analysis

The gas production values were calculated using the formula *GPt* = *Pt*×(*V*_0_ − *V*_1_)/(101.3×*W*), where *GPt* is the gas production (mL/g DM) at time point *t*, *Pt* is the pressure (mPa) at time point *t*, *V*_0_ is the volume (mL) of the serum bottle, *V*_1_ is the volume (mL of the incubation medium, 101.3 is the standard atmospheric pressure (mPa), and *W* is the weight (g) of the dried silage mixture. The cumulative gas production from each serum bottle was corrected by the gas production of the blank, as described by Contreras-Govea et al [21].

The following exponential monophasic model was chosen and fitted to corrected cumulative gas production using the GraphPad Prism v.8.02 software for windows (GraphPad Software Inc., San Diego, CA, USA): *Y* = *A*+*B*×(1−e^−^*^C^*^(^*^t^*^−^*^lag^*^)^), where *Y* is the cumulative gas production (mL/g DM) during a time period, *A* is the gas production (mL/g DM) during the rapid fermentation period, *B* is the gas production (mL/g DM) during the slow fermentation period, *C* is the constant rate of gas production (mL/h), *t* is the incubation time (h), *lag* is the initial delay before gas production (h), and *A*+*B* is the potential gas production (mL/g DM).

The effects of the LAB strains on the fermentation characteristics and *in vitro* rumen fermentation were analyzed by one-way analysis of variance using SPSS software (IBM SPSS Statistics 25) following the general linear model: Y_i_ = u+T_i_+e_i_, where Y_i_ is the dependent variable, u is the overall mean, T_i_ is the treatment effect, and e_i_ is the error term.

Regardless of the significance of treatment effect, Tukey’s multiple comparisons was used to test differences between individual means. p<0.05 were set as the significance level.

## RESULTS

### The bacterial diversity of FTMRs

**Figure 1:**
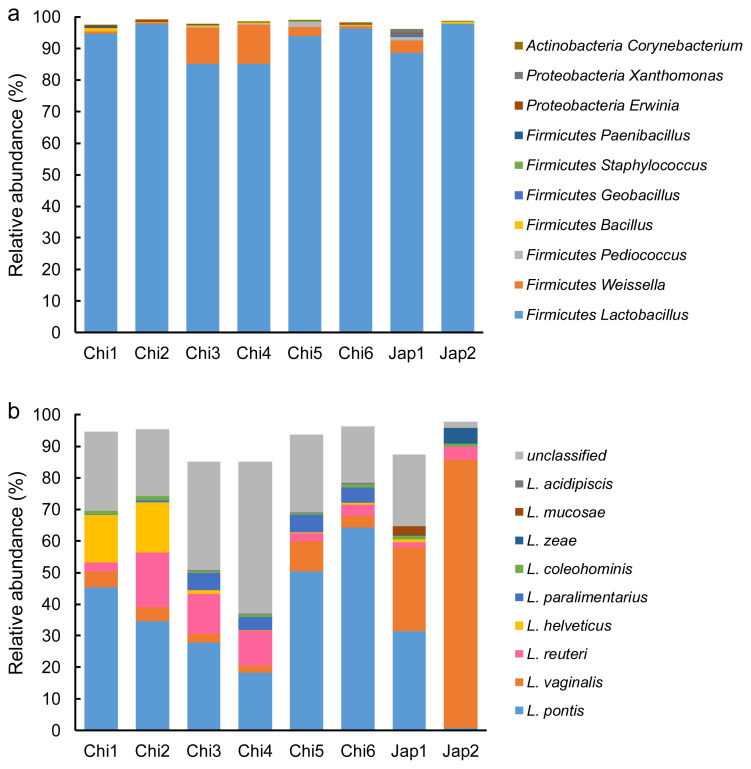
Taxonomic profiles of different silage microbial communities at different taxonomic ranks. Chi1, Chi2, Chi3, Chi4, Chi5, and Chi6 represent the different FTMRs collected from Nbdg YOYOU Company, China. Jap1 and Jap2 represent the different FTMRs collected from Japan. (a), (b) Represents bacterial relative abundance at genus (top 10), and species levels of *Lactobacillus* (top 10), respectively.

After filtering and quality control, a total of 447,730 quality-checked bacterial 16S rRNA gene sequences were obtained from the 24 FTMR samples, with an average of 18,655 sequences per sample. The Good’s coverage values were all approximately 1. *Firmicutes* was the predominant bacteria in all FTMRs, showing more than 95% relative abundance, followed by *Proteobacteria*, *Actinobacteria* and other unidentified bacteria (Figure 1a). Similar results were observed in the major bacterial genera in all FTMRs belonging to *Lactobacillus*, covering more than 85% relative abundance, while *Weissella*, *Pediococcus*, *Bacillus*, *Geobacillus*, *Staphylococcus*, *Paenibacillus*, *Erwinia*, *Xanthomonas*, and *Corynebacterium* occupied the remaining portion (Figure 1a). Nine species were observed in *Lactobacillus* in the current study: *L. pontis*, *L. vaginalis*, *L. reuteri*, *L. helveticus*, *L. paralimentarius*, *L. coleohominis*, *L. zeae*, *L. mucosae*, and *L. acidipiscis*, in which *L. pontis*, *L. vaginalis*, *L. reuteri*, *L. helveticus*, and *L. paralimentarius* showed a relative abundance of >4% in specific FTMRs (Figure 1b). The predominant species of *Lactobacillus* in Chi1, Chi2, Chi3, Chi4, Chi5, Chi6, and Jap1 was *L. pontis*, with relative abundances of 45%, 35%, 28%, 18%, 50%, 64%, and 31%, respectively. The major species in Jap2 was *L. vaginalis*, which showed a relative abundance of 85% in this sample. The relative abundances of *L. reuteri* in Chi2, Chi3, and Chi4 was 17%, 13%, and 11%, respectively. The relative abundances of *L. helveticus* in Chi1 and Chi2 were 15% and 16%, respectively. The relative abundances of *L. paralimentarius* in Chi3, Chi4, Chi5, and Chi6 were 5%, 4%, 6%, and 5%, respectively.

### Ensiling characteristics of silages

The dynamics of the pH of corn stover silages are presented in Table 1. The silage pH values of CSI, *L. helveticus*, and *L. paralimentarius* were below 4.20, beginning on day 3, and the silage pH values of CSI and *L. paralimentarius* were lower (p<0.05) than that of *L. helveticus*. The silage pH value of the other treatments was below 4.20, beginning on day 7. The pH values of all silages were higher than 4.2, on day 56, in which *L. vaginalis* was 4.57, and the others varied between 4.22 and 4.37.

The dynamics of lactic acid concentrations in corn stover silages are presented in Table 2. The lactic acid concentration increased continuously during the first 28 days to a concentration of 2.68 g/kg DM and then dropped to 1.86 g/kg DM on day 56 in the control. A similar pattern was observed in the CSI, whose concentration of lactic acid was 3.19 g/kg DM on day 14 and then decreased to 2.14 g/kg DM on day 56. However, the lactic acid concentration in this sample was higher (p<0.05) than that of the control from days 1 to 14. The lactic acid concentration of *L. paralimentarius* treatment was higher (p<0.05) than that of the control from days 1 to 14, but was higher (p<0.05) only than that of the CSI treatment on day 1, and only one time point was different (p<0.05) between *L. vaginalis* and the control, *L. reuteri* and the control, and *L. helveticus* and the control. These differences were observed on days 14, 7, and 56, respectively.

**Figure 2:**
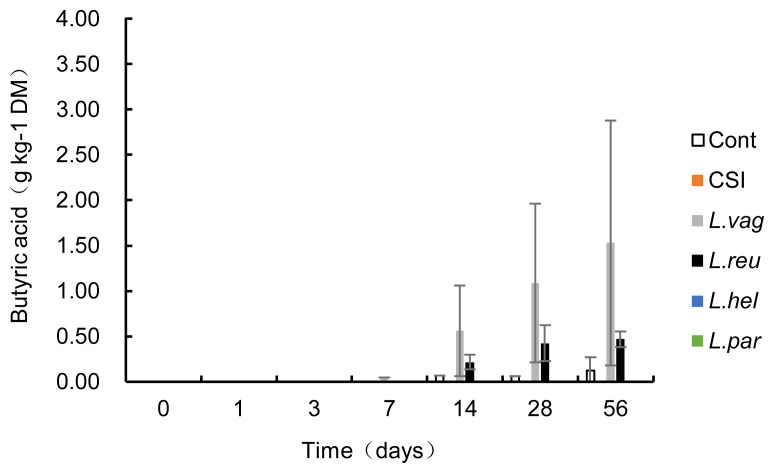
Butyric acid concentration in each treatment during the ensiling period. Cont, control; CSI, commercial silage inoculant silage; *L. vag*, *L. vaginalis* silage; *L. reu*, *L. reuteri* silage; *L. hel*, *L. helveticus* silage; *L. par*, *L. paralimentarius* silage.

The acetic acid concentration increased exponentially during ensiling, with day 7 being the turning point (Table 3). Compared to the control, all five treatments decreased (p<0.05) the acetic acid concentration during ensiling, but only *L. paralimentarius* treatment decreased (p<0.05) the acetic acid concentration during the whole fermentation. Furthermore, acetic acid in the *L. paralimentarius* group was lower (p<0.05) than that in the CSI on days 1, 7, and 28. Butyric acid was detectable after 7 days of ensiling in *L. vaginalis* and 14 days of ensiling in the control and *L. reuteri* groups (Figure 2). The butyric acid concentrations were 0.13, 1.53, and 0.47 g/kg DM in the control, *L. vaginalis*, and *L. reuteri* groups on day 56, respectively. Propionic acid, isobutyric acid, valeric acid, and isovaleric acid were undetectable in the silage.

During the ensiling period, NH _3_-N production was below 3.14 g/kg DM for all samples (Table 4). Only the *L. paralimentarius* group on day 1 and *L. reuteri* group on day 7 decreased (p<0.05) NH_3_-N production.

### *In vitro* rumen fermentation characteristics

*In vitro* rumen fermentation characteristics are presented in Table 5. Compared to the control, *L. helveticus* increased (p<0.05) the cumulative gas production of fermentation, and the other treatments had no effect on cumulative gas production. The potential gas production, pH, NH_3_-N, isobutyric acid, and isovaleric acid in the CSI treatment group were all higher (p<0.05) than those of the control, and the lag time was lower (p<0.05) than that of the control. While *L. helveticus* increased (p<0.05) potential gas production and isovaleric acid, *L. reuteri* and *L. paralimentarius* increased (p<0.05) potential gas production only.

## DISCUSSION

Investigating the diversity of LAB in silage is helpful in selecting microbial species as efficient inoculants, regardless of the ensiling origin and the object [10]. Guan et al [22] reported that after fermentation, the relative abundance of the genus *Lactobacillus* exceeded 50% in corn bunker-silo silages, and Yan et al [23] reported that the genus *Lactobacillus* showed 44.16% relative abundance in Italian ryegrass silage, 45.94% relative abundance in Italian ryegrass silage prepared with corn stover and exceeded 80% relative abundance in Italian ryegrass silage prepared with a commercial inoculant strain of *Lactobacillus plantarum*. The genus *Lactobacillus* covered the major relative abundance of silage, and *L. plantarum*, *L. acidophilus*, *L. buchneri*, and *L. hilgardii*, which promote silage quality improvement [24,25]. In the current selected FTMRs, the dominant bacteria during ensiling belonged to the phylum *Firmicute*, comprising genera *Lactobacillus*, *Lactococcus*, *Weissella*, and *Leuconostoc*, while *Lactobacillus* exceeded 85% relative abundance. *Lactobacillus* has higher acid resistance than *Lactococcus*, *Weissella* and *Leuconostoc*, thus the higher relative abundance of *Lactobacillus* in our experiment might be due to the more lactic acid produced by the diverse feed composition in FTMRs. And the selected strains in our experiment will maintain the pH of corn stover silage at very low, such as 3.55 in *L. paralimentarius*. *L. pontis*, *L. vaginalis*, *L. reuteri*, *L. helveticus*, and *L. paralimentarius* were the dominant species in eight FTMRs, but the five species had not been the focus of previous studies. To improve the accuracy of selection, the fermentation patterns must be evaluated. Furthermore, there are differences between strains of the same species for ensiling [10]. Therefore, in order to facilitate the verification and application of our results, the standard strain of *L. pontis*, *L. vaginalis*, *L. reuteri*, *L. helveticus*, and *L. paralimentarius*, identified by 16S rRNA sequence analysis of FTMRs in this study, were selected evaluating the ensiling characteristics for corn stover. However, *L. pontis* (DSM 8475) was eliminated from the list during ensiling because of its slow growth.

**Figure 3:**
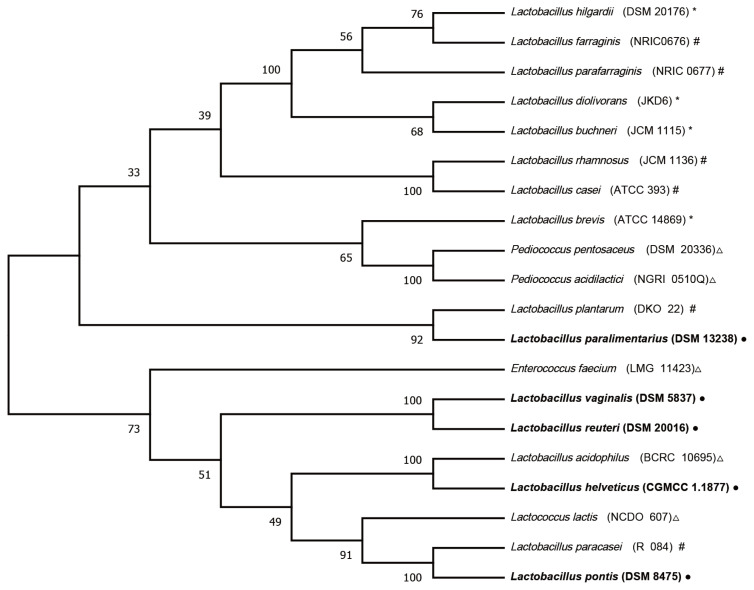
Neighbor-joining tree showing the phylogenetic tree of the lactic acid bacteria (LAB) strains studied as inoculants for silage using 16S rRNA gene sequences retrieved from the GeneBank database. Sequences were aligned with ClustalW, and the phylogenetic tree was constructed using the MEGA-X program. Δ Homofermentative; * Obligately heterofermentative; # Facultatively heterofermentative; ● LAB strains in the current study.

The LAB used as additives can be divided into homofermentative, obligately heterofermentative, and facultatively heterofermentative species according to their metabolic types. Inoculation with homofermentative or facultatively heterofermentative LAB rapidly produces lactic acid (pKa = 3.86), which is responsible for the decrease in pH values. In contrast, obligately heterofermentative LAB are mainly used to improve aerobic stability due to the production of acetic acid and propionic acid, which has an antifungal effect [1]. The phylogenetic relationships among the validated species in the current study, the main LABs [26] that have been evaluated as silage inoculants, and the LABs in CSI are shown in Figure 3. *L. vaginalis*, *L. reuteri*, and *L. helveticus* clustered together with *L. acidophilus* (BCRC 10695), whereas *L. paralimentarius* clustered together with *L. plantarum* (DKO 22), a type of *Lactobacillus* species in the CSI. These *Lactobacillus* species are homofermentative or facultatively heterofermentative species. McCullough [27] subdivided silage fermentation and storage into six phases. The last phase is the feed-out period, aerobic organisms convert lactic acid to carbon dioxide and water, thus increasing pH. Consistent with this knowledge, with access to the end phase of silage fermentation, homofermentative *Lactobacillus* species, with their acid production and adaptation abilities, established their dominant position in our current experiment. Although the epiphytic LAB naturally present in the corn stover can decrease the pH to below 4.20, beginning on day 7, homofermentative *Lactobacillus* species in the CSI and homofermentative *L. paralimentarius* accelerated this decline, which could ensure better preservation of corn stover silage than natural fermentation.

In addition to lactic acid, acetic acid and butyric acid in some treatments were detected during fermentation. Moderate concentrations of acetic acid in silage are beneficial for ensiling because they can inhibit yeasts, resulting in improved stability when the silage is exposed to air. In the current experiment, all bags were sealed after vacuuming during sampling, so the data could not be used to evaluate aerobic stability. However, intermittent sampling followed by vacuuming, undoubtedly reintroduced oxygen into the silo bag, stimulated aerobic fermentation, and the pH increased and lactic acid concentration decreased at the end of fermentation (day 56) when compared with 3 to 28 days of storage. These phenomena coupled with the increase in acetic acid production, indicate that the predominant fermentation model might have changed. One possible explanation might be that homofermentation shifted to heterofermentation during ensiling. Monitoring the changes in viable numbers of LAB, yeast, and mold, and the microbial communities during ensiling would be helpful to explain this shift in the fermentative model in the future. Butyric acid was only detected in the control, *L. vaginalis*, and *L. reuteri* silages started on days 14, 14, and 7, respectively. The presence of this acid indicates the metabolic activity of clostridia organisms. Some clostridia are able to ferment sugars to butyric acid, some can convert lactic acid to butyric acid, and some species are highly proteolytic. Higher NH_3_-N production is usually a result of proteolytic activity by clostridia. In the current study, the NH_3_-N production in all treatment groups was lower than 4 mg/g DM, which is an order of magnitude lower than that reported [22]. These results might be ascribed to the low CP and amino acid levels in corn stover, which are not sufficient to affect NH_3_-N production.

There are differences between strains of the same species during ensiling [10]. Thus, the selection of standard strains for use in silages would be easier to popularize. *L. plantarum* is one of the two homofermentative LAB of commercial silage inoculant used in the CSI. Ren et al [24] and Xu et al [28] reported that *L. plantarum* strains can modulate the bacterial community in silage by enriching *Lactobacillus* and reducing microbial richness/diversity. In addition, some *L. plantarum* strains express multifunctional glycoside hydrolases; thus, an increase in the WSC content of silage has been observed [29]. With the high similarity of the 16S rRNA gene between *L. paralimentarius* and *L. plantarum*, similar fermentation of corn stover silage was observed between the *L. paralimentarius* and CSI groups. *Pediococcus acidilactici*, another homofermentative LAB of commercial silage inoculant used in the CSI, has been reported as an inoculant and improved the quality of silage of different forages [30]. The current study supports the hypothesis that the homofermentative strain *L. paralimentarius* accelerated the decrease in pH values of corn stover silage. *L. paralimentarius* was identified in the FTMRs of Chi1 to Chi6, including soybean, corn, corn husk, wheat bran, brewer grains, dry peanut straw, and fresh rice straw. Therefore, *L. paralimentarius* should be an effective strain to improve the fermentation of corn husk, wheat bran, brewer grains, dry peanut straw, and fresh rice straw. However, ensiling legumes were not easy due to their low WSC concentration, examining the effect of *L. paralimentarius* on the fermentation of this silage is necessary to broaden its range of application in the future.

Evaluating the feeding value of silage inoculated with the LAB strain should be the last step in strain selection. *In vitro* gas measurement has become a routine method of feed evaluation and has been widely used to investigate the digestion kinetics of both soluble and insoluble fractions of feedstuffs. Therefore, this method was used in the current experiment. Using this method, we confirmed that *L. paralimentarius* inoculation had positive effects on the silage feeding value, and the rapidly fermented part of corn stover was fermented even more during ensiling, which was deduced by the low rate of gas production and the high ratio of acetic acid to propionic acid. In *in vitro* rumen fermentation measurements, *L. helveticus* and *L. reuteri* did not affect the quality of the corn stover silage, but had a positive effect on potential gas production, which might be the direct effect of LAB on rumen fermentation and should be tested in the future.

In this study, *L. paralimentarius* achieved the equal effect as commercial additive and led to higher quality of corn stover silage, which is consistent with results that it showed a marked ability to produce acid, and enhance the degradation of the corn stover. In a word, this single strain is highly efficient and benefit to reduce cost when application. The results indicated the method to test standard strain of some candidate strains selected from FTMRs could be used to efficiently screen for LAB additives for corn stover silage.

## CONCLUSION

We found that members of the genus *Lactobacillus* were the dominant bacteria in all eight FTMRs. The species that showed a relative abundance greater than 4% in at least two FTMRs included *L. paralimentarius*, *L. helveticus*, *L. reuteri*, *L. vaginalis*, and *L. pontis*. Similar to the effects of CSI, *L. paralimentarius* was highly effective in reducing the pH value and producing lactic acid in corn stover silage, which indicated that *L. paralimentarius* may be a promising ensiling inoculant for corn stover silage. Furthermore, selecting LAB strains from FTMRs may be a feasible strategy to improve the silage quality.

## Figures and Tables

**Table 1:** The pH values of each treatment during the ensiling period

Time (d)	Treatment^1)^						SEM	p-value

	Cont	CSI	*L. vag*	*L. reu*	*L. hel*	*L. par*		
0	6.33^ab^	6.26^ab^	6.29^ab^	6.44^a^	6.01^bc^	5.85^c^	0.14	<0.01
1	5.30^b^	4.45^c^	5.38^ab^	5.54^a^	5.26^b^	4.28^c^	0.07	<0.01
3	4.23^ab^	3.91^c^	4.30^a^	4.32^a^	4.14^b^	3.79^c^	0.06	<0.01
7	3.90^bc^	3.62^d^	3.98^a^	3.91^b^	3.84^c^	3.67^d^	0.03	<0.01
14	3.75^ab^	3.54^c^	3.89^a^	3.76^ab^	3.68^bc^	3.55^c^	0.05	<0.01
28	3.95	3.78	3.83	3.98	3.91	3.85	0.07	0.21
56	4.35^ab^	4.22^b^	4.57^a^	4.37^ab^	4.30^b^	4.31^b^	0.07	<0.01

SEM, standard error of the mean.

1) Cont, control; CSI, commercial silage inoculant silage; *L. vag*, *L. vaginalis* silage; *L. reu*, *L. reuteri* silage; *L. hel*, *L. helveticus* silage; *L. par*, *L. paralimentarius* silage.

a–d Means in the same row with different superscripted letters differ (p<0.05).

**Table 2:** Lactic acid concentrations of each treatment during the ensiling period (g/kg dry matter)

Time (d)	Treatment^1)^						SEM	p-value

	Cont	CSI	*L. vag*	*L. reu*	*L. hel*	*L. par*		
0	0.09	0.10	0.04	0.00	0.02	0.12	0.06	0.14
1	0.42^c^	1.26^b^	0.37^c^	0.31^c^	0.67^c^	2.77^a^	0.16	<0.01
3	1.23^c^	1.85^ab^	1.14^c^	1.05^c^	1.46^bc^	2.17^a^	0.20	<0.01
7	1.85^b^	2.34^a^	1.60^bc^	1.44^c^	1.79^b^	2.36^a^	0.13	<0.01
14	2.40^b^	3.19^a^	1.67^c^	2.03^bc^	2.25^bc^	3.08^a^	0.26	<0.01
28	2.68^abc^	3.15^a^	1.53^c^	2.32^bc^	2.66^abc^	2.85^ab^	0.24	<0.01
56	1.86^b^	2.14^b^	2.29^b^	2.33^b^	3.08^a^	1.88^b^	0.26	<0.01

SEM, standard error of the mean.

1) Cont, control; CSI, commercial silage inoculant silage; *L. vag*, *L. vaginalis* silage; *L. reu*, *L. reuteri* silage; *L. hel*, *L. helveticus* silage; *L. par*, *L. paralimentarius* silage.

a–c Means in the same row with different superscripted letters differ (p<0.05).

**Table 3:** Acetic acid concentrations of each treatment during the ensiling period (g/kg dry matter)

Time (d)	Treatment^1)^						SEM	p-value

	Cont	CSI	*L. vag*	*L. reu*	*L. hel*	*L. par*		
0	0.00	0.00	0.00	0.00	0.00	0.00	-	-
1	1.20^a^	1.22^a^	1.18^a^	1.15^a^	1.11^a^	0.24^b^	0.11	<0.01
3	1.80^ab^	1.53^bc^	2.13^a^	2.11^a^	1.79^ab^	1.22^c^	0.12	<0.01
7	3.78^a^	3.18^b^	3.58^ab^	3.44^ab^	3.25^b^	2.74^c^	0.16	<0.01
14	4.22^a^	3.57^bc^	3.73^b^	3.97^ab^	3.57^bc^	3.11^c^	0.18	<0.01
28	4.12^a^	3.66^a^	3.67^a^	4.16^a^	3.91^a^	3.03^b^	0.20	<0.01
56	5.04^a^	3.90^bc^	4.07^abc^	4.65^bc^	4.14^abc^	3.06^c^	0.40	<0.01

SEM, standard error of the mean.

1) Cont, control; CSI, commercial silage inoculant silage; *L. vag*, *L. vaginalis* silage; *L. reu*, *L. reuteri* silage; *L. hel*, *L. helveticus* silage; *L. par*, *L. paralimentarius* silage.

a–c Means in the same row with different superscripted letters differ (p<0.05).

**Table 4:** NH_3_-N production of each treatment during the ensiling period (mg/g dry matter)

Time (d)	Treatment^1)^						SEM	p-value

	Cont	CSI	*L. vag*	*L. reu*	*L. hel*	*L. par*		
0	0.81	0.92	0.74	0.65	0.70	0.84	0.22	0.4
1	1.26^a^	1.09^ab^	1.20^a^	1.18^a^	1.10^ab^	0.94^b^	0.13	<0.01
3	1.39	1.30	1.35	1.40	1.29	1.15	0.21	0.31
7	1.58^a^	1.23^ab^	1.25^ab^	1.14^b^	1.25^ab^	1.19^ab^	0.21	0.02
14	2.04	2.07	2.05	1.81	2.07	2.16	0.39	0.67
28	3.14	2.84	2.54	2.92	2.92	2.76	0.35	0.12
56	1.83	1.69	1.96	1.76	1.84	1.75	0.25	0.47

SEM, standard error of the mean.

1) Cont, control; CSI, commercial silage inoculant silage; *L. vag*, *L. vaginalis* silage; *L. reu*, *L. reuteri* silage; *L. hel*, *L. helveticus* silage; *L. par*, *L. paralimentarius* silage.

a,b Means in the same row with different superscripted letters differ (p<0.05).

**Table 5:** *In vitro* rumen fermentation characteristics

Item	Treatment^1)^						SEM	p-value

	Cont	CSI	*L. vag*	*L. reu*	*L. hel*	*L. par*		
Cumulative GP_72h_ (mL/g DM)	84.49^b^	104.37^ab^	94.91^b^	100.16^ab^	118.19^a^	91.21^b^	3.52	0.06
Potential GP (mL/g DM)	94.79^c^	112.08^ab^	105.59^bc^	112.75^ab^	127.21^a^	125.63^a^	3.23	<0.01
Rate of GP (mL/h/g DM)	0.032^a^	0.038^a^	0.031^a^	0.031^a^	0.036^a^	0.020^b^	<0.01	<0.01
Lag time (h)	4.35^ab^	3.55^b^	4.37^ab^	5.32^a^	4.74^ab^	5.20^a^	0.22	0.17
pH_72h_	7.02^bc^	7.14^a^	7.06^b^	6.99^c^	7.03^bc^	7.06^b^	0.01	<0.01
NH_3_-N (mg/g DM)	14.40^b^	17.89^a^	16.59^ab^	15.55^ab^	15.96^ab^	15.99^ab^	0.34	0.06
MCP (mg/g DM)	18.69	20.52	18.77	18.46	18.36	18.75	0.31	0.38
Acetic acid (mmol/L)	13.15	14.18	13.59	14.26	15.85	12.60	0.46	0.46
Propionic acid (mmol/L)	5.46	6.48	5.37	5.34	6.63	4.82	0.26	0.29
Butyric acid (mmol/L)	1.57^ab^	2.21^a^	1.63^ab^	1.54^b^	2.08^ab^	1.53^b^	0.10	0.31
Isobutyric acid (mmol/L)	0.23^b^	0.29^a^	0.27^ab^	0.24^ab^	0.27^ab^	0.25^ab^	0.01	0.13
Valeric acid (mmol/L)	0.23	0.26	0.24	0.23	0.27	0.25	0.01	0.15
Isovaleric acid (mmol/L)	0.28^b^	0.37^a^	0.33^ab^	0.29^ab^	0.37^a^	0.3^ab^	0.01	0.46
Total VFA (mmol/L)	20.92	23.79	21.43	21.89	25.46	19.75	0.79	0.40
Acetic acid:propionic acid molar ratio	2.46^ab^	2.19^b^	2.55^a^	2.72^a^	2.39^ab^	2.62^a^	0.06	0.06

SEM, standard error of the mean; GP, gas production; DM, dry matter; MCP, microbial crude protein; VFA, volatile fatty acids.

1) Cont, control; CSI, commercial silage inoculant silage; *L. vag*, *L. vaginalis* silage; *L. reu*, *L. reuteri* silage; *L. hel*, *L. helveticus* silage; *L. par*, *L. paralimentarius* silage.

a–d Means in the same row with different superscripted letters differ (p<0.05).
